# Acute Adrenal Hemorrhage as the Initial Manifestation of Metastatic Lung Adenocarcinoma: A Cautionary Tale

**DOI:** 10.1155/2022/2864773

**Published:** 2022-10-25

**Authors:** Sowbharnika Arivazhagan, Guru Prasad Parthiban, Christina Patterson, Karthik Reddy

**Affiliations:** Baton Rouge General Medical Center, 8585 Picardy Avenue, Baton Rouge 70809, USA

## Abstract

Adrenal gland metastatic disease is the most commonly occurring malignancy of the adrenal glands. Although metastatic disease is common, adrenal hemorrhage is a rare but potentially fatal manifestation of malignancy. The objectives of this case report are to highlight the unusual presentation of metastatic lung adenocarcinoma as spontaneous adrenal hemorrhage in a 64-year-old female who was otherwise asymptomatic. As well as to support the reasoning that metastatic disease should be considered as a differential in patients with this clinical presentation as this may have altered this fatal outcome.

## 1. Introduction

Lung cancer is the second most common cancer diagnosis in the United States. Non-small lung cancer accounts for approximately 85% of lung cancers [1]. Per USPSTF recommendations, annual screening for lung cancer with low dose CT should begin in patients aged 50 who have a 20-pack year history and currently smoking or have quit in the past 15 years. But, when screenings have not been performed, most patients who present with symptoms due to lung cancer most likely have advanced disease. The adrenal gland is a common site for metastasis of non-small cell carcinoma but an uncommon initial manifestation [2]. Approximately, 10% of patients diagnosed with lung cancer will develop adrenal metastasis. Only 2 to 3% of these occur at the initial presentation of non-small cell lung cancer. Of four hundred and sixty-four patients with metastatic disease in the adrenal glands included in one study, 94% of the metastatic adrenal tumors were carcinomas and 56% of these were adenocarcinoma [3]. Adrenal hemorrhage is a rare disorder, estimated to be present in 0.14% to 1.8% of postmortem examinations and is associated with a 15% mortality rate [4]. Patients can present with nonspecific symptoms of abdominal pain, nausea, lethargy, weakness, and malaise. We report on a 64-year-old Caucasian female who presented for acute left upper quadrant abdominal pain and was found to have left adrenal hemorrhage secondary to adenocarcinoma metastasis.

## 2. Case Presentation

**Figure 1:**
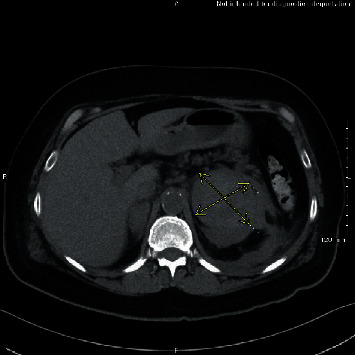
Demarcation of Left adrenal hemorrhage.

**Figure 2:**
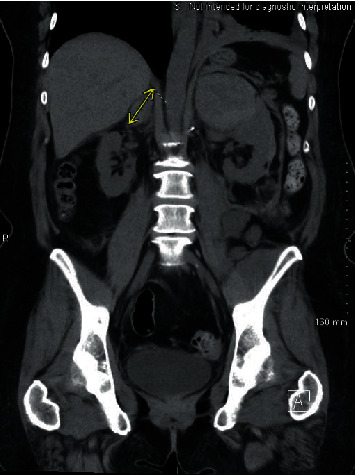
Demarcation of right adrenal hemorrhage.

We present a 64-year-old Caucasian female with 35 pack-year smoking history and no comorbidities who initially presented to an outside facility due to acute left upper quadrant abdominal pain. She was eventually diagnosed with acute left adrenal hemorrhage, transfused packed red blood cells (PRBC), and underwent embolization of the left adrenal artery. She was later discharged to follow up with her PCP. A few days later, she presented to our hospital with complaints of shortness of breath (SOB) with exertion. The patient reported a three-week history of SOB prior to presentation, which worsened following the recent hospital discharge. She was hemodynamically stable upon presentation. Physical examination was significant for generalized pallor, jaundiced mucosa, and a large hematoma surrounding the left flank. The patient's hemoglobin on presentation was 7.5 (7.2 at discharge from the previous hospital). She also had severe orthostatic hypotension. Her other labs include urea 16, creatinine 0.89, sodium 136, potassium 4, AST 90, ALT 62, ALP 69, total bilirubin 3.4, WBC 7.6, platelets 97000, and cortisol > 20. Her chest X-ray revealed a small pleural effusion on the left, which was initially thought to be a sympathetic effusion from underlying hematoma. CT Angiogram of abdomen and pelvis revealed evidence of coiling of the left adrenal gland with a 6 cm mass likely representing hemorrhage in the left adrenal gland itself. There was a hemorrhage in the adjacent pararenal space crossing the midline in the retroperitoneum in the right paracolic gutter without evidence of active bleeding. She was admitted for further workup of her progressively worsening symptoms and etiology of the adrenal hemorrhage. Despite adequate volume resuscitation with PRBC and IV fluids, the patient remained symptomatic with no significant improvement in blood count. A repeat chest X-ray was obtained which revealed worsening of the pleural effusion, hence the patient underwent thoracentesis which revealed bloody exudative effusion (>2 million RBC) but cytology did not reveal malignant cells. Due to high suspicion of ongoing malignancy, repeat imaging was obtained. CT abdomen and pelvis revealed worsening left hematoma with additional new right adrenal hemorrhage (Figures 1 and 2). CT chest revealed scattered lymphadenopathy in the left supraclavicular neck, bilateral axilla, and left hilum with questionable metastatic adenopathy, along with a small indeterminate 5 mm left upper lobe pulmonary nodule. MRI of the brain revealed multiple scattered tiny foci concerning a metastatic disease. Eventually, the patient underwent an excisional biopsy of the right axillary lymph node, with the hope of identifying the primary tumor. While awaiting pathology reports, we also ruled out tuberculosis with QuantiFERON Gold and autoimmune etiology with an extensive workup. The plan was to evaluate the extent of the disease with imaging once pathology was available, and plan cancer treatment accordingly.

Unfortunately, the patient clinically deteriorated due to the development of disseminated intravascular coagulation (DIC). Her renal function worsened due to acute tubular necrosis related to DIC, intravascular volume depletion, and hypotension. Mentation gradually deteriorated due to uremic encephalopathy, and renal replacement therapy was planned. Following vascular catheter insertion, the patient had a large pneumothorax with hemodynamic compromise and had to be transferred to the ICU. Despite the reversal of the pneumothorax, her hemodynamics did not improve with vasopressor refractory shock and the patient succumbed to death. We received the final pathology result after her demise as “Poorly differentiated adenocarcinoma, primary lung cancer.”

## 3. Discussion

Adrenal hemorrhage is rare but potentially lethal. Clinical manifestations can range from subclinical unilateral hemorrhage to massive bilateral hemorrhage leading to cardiovascular collapse and death [5]. Possible etiologies include anticoagulation, sepsis, bleeding disorders, metastatic disease, or trauma [6]. Among metastatic diseases, the lung, GI tract, breast, and kidney are the most common primary tumors [7]. Incidence is more common among non-small cell lung cancer with 40% of patients developing adrenal metastasis (both unilateral and bilateral) as cancer progresses [8]. Despite its high incidence, adrenal metastasis remains asymptomatic. The incidence of adrenal metastasis being the initial presentation of primary lung cancer is extremely low, and symptomatic metastasis with hemorrhage can be found only in a few cases reported in English literature [2]. The signs and symptoms of adrenal hemorrhage are usually nonspecific including abdominal, chest or back pain, nausea, vomiting, hypotension, tachycardia, and fever [9]. The etiology of tumor-related adrenal hemorrhage is multifactorial. One of the mechanisms proposed includes tumor expansion with increased vascularity due to upregulation of fibroblast growth factor, interleukin 8, vascular endothelial growth factor, and matrix metalloproteases [2].

CT and MRI remain the most sensitive and specific diagnostic modalities of adrenal hemorrhage [10]. CT scan is highly sensitive in detecting the nature of adrenal metastasis, but benign adenomas could be mistaken as metastasis [11]. MRI is superior to CT scan in differentiating adenomas from metastasis [12], also in differentiating subacute/acute hemorrhage from chronic ones [6]. CT guided percutaneous biopsy of the adrenal mass is a highly specific (99% specificity) diagnostic tool that is safe and reliable as well (potential complication 2.8%) [11].

Adrenal metastasis with hemorrhage can be managed with surgery, blood transfusion, chemotherapy, steroid pulse, or arterial embolism depending on the severity [13]. In severe adrenal hemorrhage that is refractory to blood transfusion and conservative measures, embolization of the bleeding adrenal artery through an endovascular approach can be lifesaving [6]. If adrenal metastasis represents the solitary site of metastasis with a resectable primary lung tumor, adrenalectomy with primary lung tumor resection improves overall survival [12]. Complete tumor resection may not be feasible if the adrenal mass is surrounded by a very large retroperitoneal hematoma. In such cases, transarterial embolization for hemodynamic stability followed by surgical resection at a later date is a possible treatment strategy [8]. Further treatment of cancer depends on staging, tumor burden, ECOG status, histology of the tumor, etc.

Despite various treatment options available as mentioned, delay in detecting the etiology of the hemorrhage may result in catastrophic outcomes. In our patient, who was healthy with no comorbidities before the adrenal hemorrhage, a meticulous search for etiology with metastatic cancer in mind during the initial presentation may have altered the clinical outcome.

## 4. Conclusion

Adrenal hemorrhage secondary to metastatic cancer is a clinical emergency. Through this article, we aim to increase awareness among clinicians. In patients who present with acute adrenal hemorrhage, stabilization of the hemorrhage with a simultaneous search for etiology should be carried out immediately to avoid unfavorable outcomes.

## Data Availability

All data generated or analyzed during this study are included in this article. Further inquiries can be directed to the corresponding author.

## References

[1] Julian R. (2008). Non-small cell lung cancer: epidemiology, risk factors, treatment, and survivorship. *Treatment and Survivorship*.

[2] Hiroi N., Yanagisawa R., Yoshida-Hiroi M. (2006). Retroperitoneal hemorrhage due to bilateral adrenal metastases from lung adenocarcinoma. *Journal of Endocrinological Investigation*.

[3] Lam K. Y., Lo C. Y. (2002). Metastatic tumours of the adrenal glands: a 30-year experience in a teaching hospital. *Clinical Endocrinology*.

[4] Mehmood K. T., Sharman T. (2020). *Adrenal hemorrhage*.

[5] Simon D. R., Palese M. A. (2009). Clinical update on the management of adrenal hemorrhage. *Current Urology Reports*.

[6] Marti J. L., Millet J., Sosa J. A., Roman S. A., Carling T., Udelsman R. (2012). Spontaneous adrenal hemorrhage with associated masses: etiology and management in 6 cases and a review of 133 reported cases. *World Journal of Surgery*.

[7] Ambika S., Melton A., Lee D., Hesketh P. J. (2009). Massive retroperitoneal adrenal hemorrhage secondary to lung cancer metastasis treated by adrenal artery embolization. *Clinical Lung Cancer*.

[8] Sahasrabudhe N., Byers R. (2009). Massive haemorrhagic adrenal metastases leading to sudden death: a case report. *Case Reports*.

[9] Wang J., Packer C. D. (2014). Acute abdominal pain after intercourse: adrenal hemorrhage as the first sign of metastatic lung cancer. *Case Reports in Medicine*.

[10] Oo T. H., Martin L., Hesketh P. J. (2002). Adrenal hemorrhage secondary to metastasis from lung cancer. *Clinical Lung Cancer*.

[11] Karanikiotis C., Tentes A. A., Markakidis S., Vafiadis K. (2004). Large bilateral adrenal metastases in non-small cell lung cancer. *World Journal of Surgical Oncology*.

[12] Singh N., Madan K., Aggarwal A. N., Das A. (2013). Symptomatic large bilateral adrenal metastases at presentation in small-cell lung cancer: a case report and review of the literature. *Journal of Thoracic Disease*.

[13] Han H., Qiao P., Jiang X. W., Wang B., Zhang X. D. (2018). Acute flank abdominal pain as the chief complaint of spontaneous adrenal hemorrhage secondary to metastatic lung cancer. *Urology Case Reports*.

